# Interaction of Te and Se interlayers with Ag or Au nanofilms in sandwich structures

**DOI:** 10.3762/bjnano.10.22

**Published:** 2019-01-21

**Authors:** Arkadiusz Ciesielski, Lukasz Skowronski, Marek Trzcinski, Ewa Górecka, Wojciech Pacuski, Tomasz Szoplik

**Affiliations:** 1 University of Warsaw, Faculty of Physics, Pasteura 5 Str., 02-093 Warsaw, Poland; 2 UTP University of Science and Technology, Institute of Mathematics and Physics, Kaliskiego 7 Str., 85-796 Bydgoszcz, Poland,; 3 University of Warsaw, Department of Chemistry, Pasteura 1 Str., 02-093 Warsaw, Poland

**Keywords:** diffusion, gold, microstrain, nanocrystallinity, permittivity, plasmonics, segregation, selenium, silver, tellurium

## Abstract

Noble metal nanolayers on flat substrates are often deposited with the use of semiconductor interlayers, which may strongly interact with the noble metal overlayer. We investigated the crystallinity, atomic concentration profile and optical parameters of ≈35 nm-thick silver and gold layers deposited on glass substrates with 2 nm-thick tellurium or selenium interlayers. Our study, based on X-ray reflectometry (XRR), X-ray diffraction (XRD), X-ray photoelectron spectroscopy (XPS) and ellipsometric measurements, showed that using either of these interlayers introduces strain in nanocrystals of both plasmonic films. This, in turn, influences the migration of Se and Te into the metal layers. Selenium atoms migrate both in the silver and gold nanolayers, while tellurium atoms migrate only in silver. The Te concentration curve clearly suggests that this migration is an effect of the segregation of Te atoms in the silver structure. The differences in crystallinity, as well as the migration process, strongly influence the optical parameters of Ag and Au. In the permittivity of Ag deposited on either Te or Se, additional plasmonic bands originating from grain boundary segregation or diffusion occur, while for the Au layer, such resonances were not pronounced. In the permittivity of both materials, the intensity of the interband transition peaks is strongly altered, possibly due to the nano-alloy formation, but more likely due to the microstrain on metal grains.

## Introduction

In recent years, there has been growing interest in layered, sandwich-like, metal–semiconductor structures, where the thickness of a single film is in the nanometer range. Such structures exhibit several interesting properties: reduced scattering losses due to the smoothened surface of the metal layer [1–6], highly controllable effective optical parameters [7–8], enhanced abrasive properties of metal films [9] as well as effects like charge transport anisotropy [10] and giant magneto-optical Kerr response [11]. We have recently shown that depositing silver with an ultrathin germanium interlayer alters the density profile of the silver film [12].

Among the most common sublayers for silver and gold are Cr [1–2], Ti [2,13–14], Ni [14–15], Cu [16], Ge [3,14–15,17] as well as amorphous hydrogenated carbon (a-C:H) [4], polymer layers [18–19] and recently also Al_2_O_3_ [9]. The use of these materials is relatively inexpensive and is an easy way to promote the adhesion of plasmonic metals to almost any ultrasmooth substrate. However, most of the aforementioned elements migrate inside the metal structure as a result of either grain boundary diffusion or segregation [20–21]. This deteriorates both the optical and electrical properties of the plasmonic layers.

The migration of the sublayer atoms inside a plasmonic layer was first discovered by Majni et al. [22], as the interdiffusion process of Au and Cr films deposited on silicon substrates. Ten years later, Wachs et al. [23] showed that when silver layers are deposited on top of germanium, Ge atoms migrate through the silver towards its surface, which they interpreted as segregation. In 2001, a similar discovery was made for Ag layers grown on Cu [24]. Since then, the effects of this phenomenon have attracted little attention. In 2014, Stefaniuk et al. [14] observed that Ag thin layers on top of Ge wetting films have a sheet resistance approximately twice higher than similar structures deposited on top of Ti or Ni sublayers. Ellipsometric and XPS measurements by Wróbel et al. [25] have shown that this increase in ohmic losses is most likely a result of Ge atoms segregating towards the surface of the silver layer. Recently, a number of works have expanded on that research reporting on additional bands in the permittivity spectrum of silver and gold layers with Ge atoms segregated in them [12,26–28]. The nature of these bands is believed to be plasmonic – metal nanograins surrounded by semiconductor atoms essentially act as nanoparticles which absorb light due to localized plasmon excitation [25–26]. If that is the case, such additional bands should be observable in the permittivity of any plasmonic metal thin layer film in which a semiconductor segregates.

Of the semiconductors, only Ge and Si have been reported to segregate in Ag thin layers [29]. However, other elements, like selenium and tellurium have been reported to segregate in bulk silver [30–32]. Therefore it is possible for similar phenomena to occur in the thin, sandwich-like structures. By segregating through the metal grain boundaries towards the surface of the metal film, Se or Te atoms can induce additional plasmonic absorption bands in the permittivity of Ag and Au. Controlling this process could allow for engineering of the optical parameters of noble metals as well as enhanced nonlinear effects [33]. Moreover, the segregation of Se and Te atoms through the silver or gold layers is one of the most promising alternatives for fabricating 2D selenium (selenene) and tellurium (tellurene) in a similar way that germanene and silicene were fabricated by Kurosawa et al. [29]. This is important due to unusual optical effects in such structures, particularly in the case of selenium [34–35].

Segregation of semiconductor atoms in plasmonic metals is mainly driven by the specific atomic interactions which cause the difference in matrix surface energies with and without the solute as well as the specific heat of mixing [21,32], which contribute to the enthalpy (Δ*H*) of segregation. However, as shown in the previous work, the segregation process can be strongly influenced by the grain size distribution of the metal layer as well as its density profile [12], which may direct the segregation towards a specific interface, accelerate it or inhibit it. This is because of the change in the entropic contribution (Δ*S*) to the Gibbs free energy (Δ*G* = Δ*H* − *T*Δ*S,* where *T* stands for temperature) of segregation. Voids at the intersection of multiple grain boundaries have a greater number of both substitutional and interstitial lattice sites accessible to the minority atom as well as higher coordination number. Therefore, a system in which minority atoms reside in such voids has a higher entropy *S* (and thus lower free enthalpy *G*) than a system in which they reside in a simple grain boundary. Therefore, the distribution of developed voids, which is linked with the density profile, may strongly influence the segregation characteristics. Here, we report on XRD and XRR measurements to investigate the crystallinity of Ag and Au nanolayers deposited on SiO_2_ substrates with 2 nm-thick Te or Se interlayers. XPS allowed us to examine the Se and Te concentration profiles in order to verify whether these elements migrate into the plasmonic metal structure. With ellipsometric measurements, we determined the permittivity of the investigated layers.

## Results and Discussion

### Influence of Te and Se on the crystallinity of the metal films

**Figure 1:**
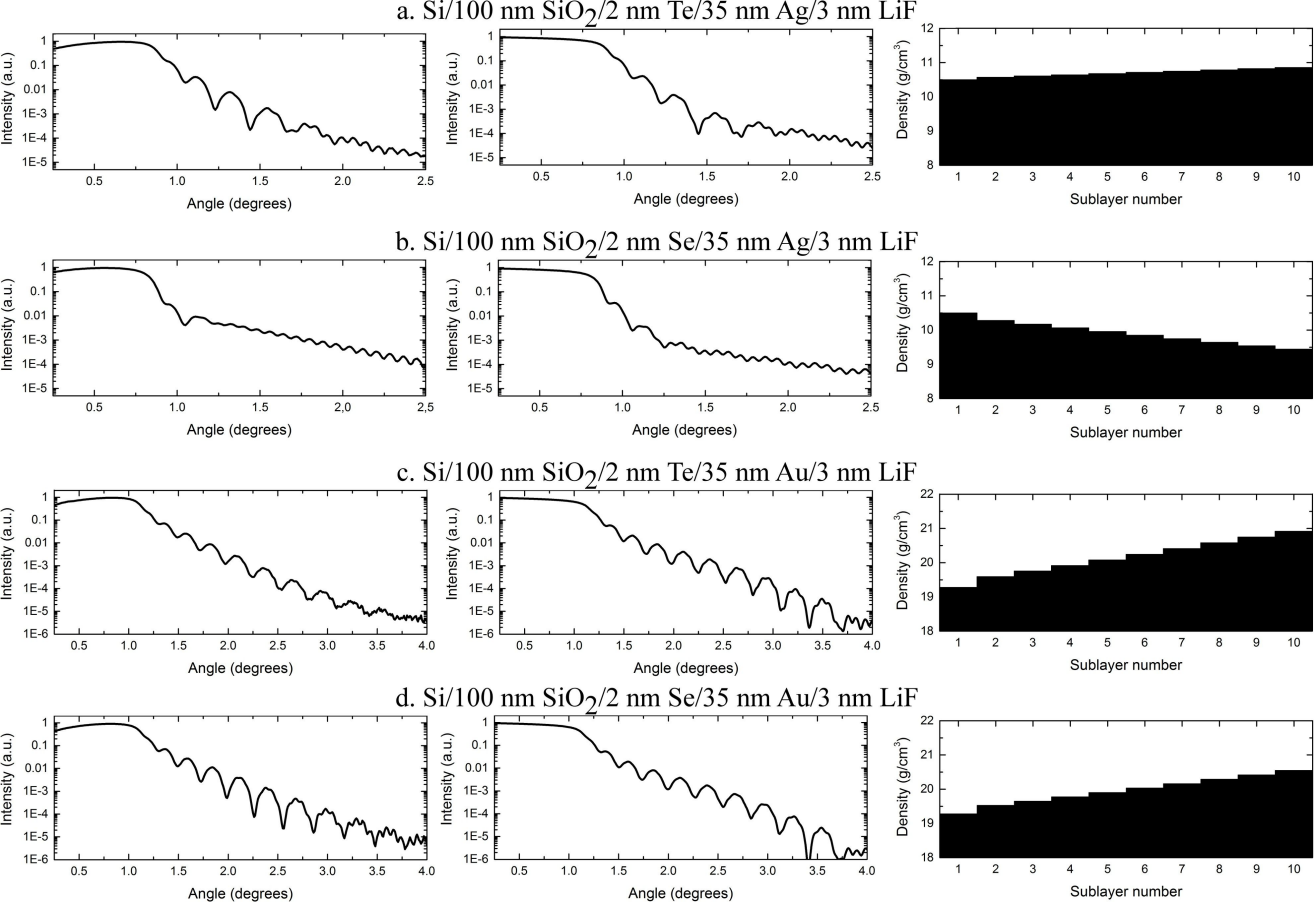
Measured XRR spectra (left column), fitted curves (middle column) as well as silver or gold layer density profiles extracted from the modeled curves (right column) for 35 nm-thick Ag or Au layers deposited on SiO_2_ with 2 nm-thick Te or Se interlayers. The Ag and Au layers in the model were divided into ten sublayers and the density slope was set to exponential. Sublayer 1 is the one at the SiO_2_/metal interface, while sublayer 10 is the one at metal/LiF interface. The oscillations with the shortest period come from the 100 nm-thick SiO_2_ substrate and are not related to the metal layers.

Table 1 shows the XRD-derived average grain size and lattice constant values of 35 nm-thick silver and gold layers deposited on SiO_2_ substrates with 2 nm-thick Te and Se interlayers, while Figure 1 shows the XRR spectra and extracted density profiles of these multilayers. Neither Te nor Se are good wetting films for silver and gold. Although the plasmonic layers deposited on Te films have their grains size decreased, the XRR extracted layer density profiles show that the density of a layer increases with increasing distance from the SiO_2_/metal interface, which is the opposite of the case of Ge-wetted films reported previously [12,28]. This indicates that the adhesion of the investigated plasmonic metals to Te is very poor. It is also worth noting that the main oscillations in the XRR spectra, related to the thickness of the metal layer, decay much faster than for samples deposited with a Ge interlayer – they cannot be observed at angles greater than 2° for silver films and 4° for gold films. This indicates a much higher surface roughness than for the Ge-wetted films. Metal layers deposited on Se exhibit an even worse adhesion – there is almost no change in the grain size, and the oscillations in the XRR spectra decay even faster in the case of silver films – they cannot be observed at angles greater than even 1.25°. Such poor adhesion should not promote the migration of Te or Se towards the surface of the metal, even if the specific interactions of those elements to silver and gold are strong. Increasing density profiles as well as large deviations of the lattice constant from sample to sample suggest an anisotropic microstrain on the metal grains. This would indicate the greatest number of nanocrystalline structure defects – and thus the greatest number of lattice sites available for semiconductor atoms to occupy [20–21] – precisely at the metal/semiconductor interface. Since such a system already has a high configurational entropy, and migration of semiconductor atoms towards the surface of the metal is unlikely to increase it. Therefore, the decrease in the Gibbs free energy of such migration is limited. That said, diffusive contributions to Δ*G* (e.g., negative heat of mixing) may induce such migration.

**Table 1:** XRD results. XRD-determined average grain size and lattice constant for 35 nm-thick Ag and Au layers deposited with 2 nm-thick Te or Se sublayers. For comparison, the values for films deposited directly on glass substrates are also presented from [12,28].

Sample	Grain size [nm]	Lattice Constant [Å]

SiO_2_/35 nm Ag/3 nm LiF	18	4.084
Si/100 nm SiO_2_/2 nm Te/35 nm Ag/3 nm LiF	12	4.078
Si/100 nm SiO_2_/2 nm Se/35 nm Ag/3 nm LiF	18	4.076
SiO_2_/35 nm Au/3 nm LiF	30	4.080
Si/100 nm SiO_2_/2 nm Te/35 nm Au/3 nm LiF	16	4.072
Si/100 nm SiO_2_/2 nm Se/35 nm Au/3 nm LiF	27	4.071

### Segregation of Te and Se into the metal structure and influence on the permittivity

**Figure 2:**
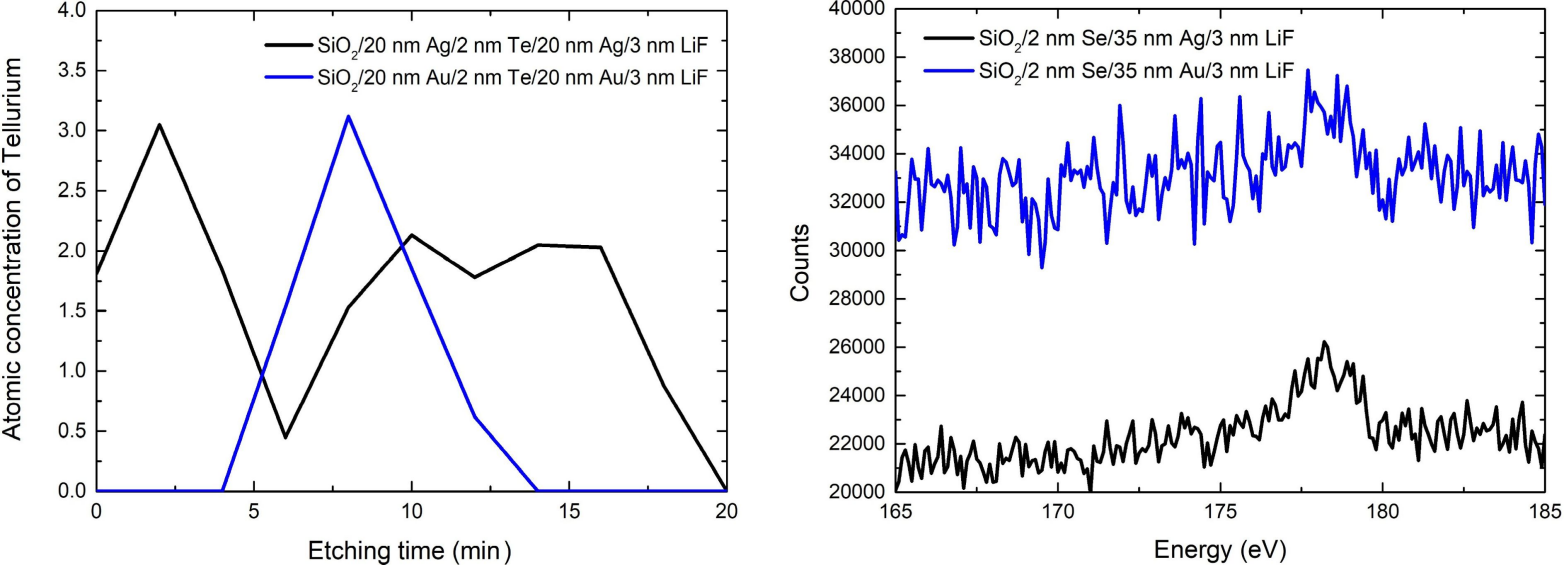
Left - Atomic concentration of Te atoms in the sandwich-like silver sample (black curve) as well as in the sandwich-like gold sample (blue curve). The etching time is equivalent to subsurface depth. Right – XPS spectra of 35 nm-thick Ag and Au layers deposited on top of 2 nm-thick Se films.

To investigate the semiconductor migration process we have fabricated sandwich-like structures at which 2 nm of Te or Se was deposited on top of 20 nm Ag or Au and then covered with another 20 nm of the same metal. Then the sample was stored for 20 days in order to allow for the diffusion and segregation progress. Then, XPS measurements interlaced with Ar-ion etching allowed for measurement of the atomic concentration of Te in such structures as a function of etching time, which is equivalent to the subsurface depth. The results for Te are presented in Figure 2 (left). A very high concentration of tellurium around the seventh to eighth minute of etching, which is basically the middle of the sample (and thus the depth where 2 nm of Te was deposited), as well as no concentration of Te at any metal/dielectric interface indicates that tellurium did not segregate nor diffuse into the gold film. In case of the silver sandwich, however, a noticeable concentration of Te can be found on both the Ag/substrate as well as the Ag/LiF interface. This could be a result of diffusion, however, in that case, the concentration of Te atoms should be equal within the whole sample (except for the very top surface, at which LiF as well as adventitious carbon and oxygen compromise the results) or at least the greatest at the middle of it. This is not the case since there is a profound dip after 6–7 minutes of etching. This indicates that tellurium has indeed segregated (and not diffused) into the silver structure. However, unlike germanium, which segregates only towards the surface of the Ag layer grown on Ge [26], Te segregates towards both Ag/dielectric interfaces. This is probably due to the fact that in a sandwich-like sample, Ge is surrounded by two different Ag layers – the one below it has flat density profile, while the one on top has a gradient density profile. This directs the segregation of Ge atoms towards the surface of the film deposited on top [12]. Tellurium, however, is surrounded by two very similar layers – both have almost flat density profile (Figure 1a). With two similar interfaces to migrate to, and similar crystalline structure of both, there is no implicit benefit to prefer one of the interfaces over the other, and thus, Te atoms segregate towards both of them.

We do not report on the concentration curves of similar structures containing selenium, since the XPS signal from selenium is very weak. The relative sensitivity factor (RSF) of the main selenium line 3d is over 13 times smaller than the RSF of the 3d line of tellurium. This means that small concentrations of selenium are much less detectable by the XPS, therefore Auger electron spectroscopy has to be used. The case of layers containing mostly silver or gold is even more difficult because the Se 3d line overlaps with both the Au 5p_3/2_ and Ag 4p_3/2_ lines. Therefore we used the Se_LMM_ Auger line as an indication of the presence of selenium. Determining the Se concentration within the sample is prone to high errors and quantitative analysis is problematic. Figure 2 (right), presents the Auger Se_LMM_ spectra collected from the very top of the surface of the metal nanolayers deposited on Se interlayer. Although the presence of this band centered at 178 eV is clearly noticeable, particularly in the case of the silver sample, it is still very weak with respect to the noise. Therefore it is hard to tell, whether this is the result of grain boundary segregation or grain boundary diffusion of selenium in the metal.

**Figure 3:**
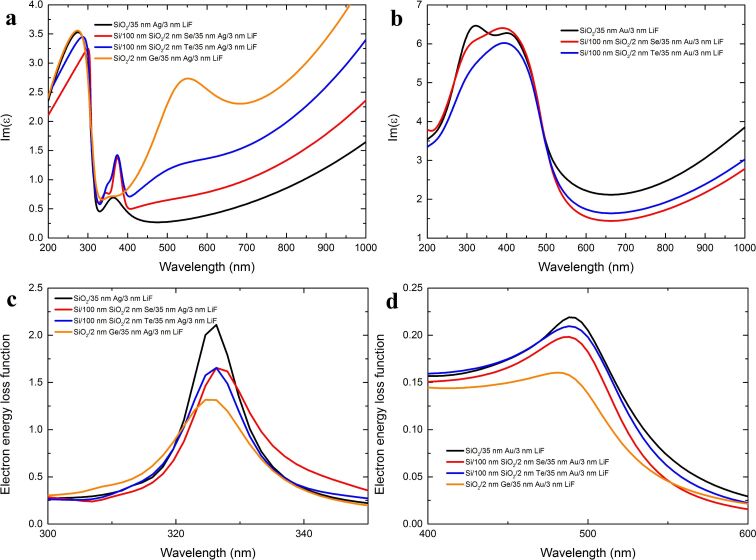
(a,b) Imaginary part of the permittivity for 35 nm-thick silver (a) and gold (b) layers deposited on Se (red curves) and Te (blue curves) interlayers. (c,d) Electron energy loss function calculated using the permittivity values presented in a and b, respectively, for wavelengths in the vicinity of the metal plasma frequency. For comparison, the curves for layers deposited directly on glass substrates (black curves) as well as Ge-wetted layers (orange curves) are also provided [26,28].

Figure 3 presents the imaginary parts of permittivity and electron energy loss function values for all of the investigated 35 nm-thick layers measured two weeks after deposition as well as for non-wetted and Ge-wetted silver and gold layers measured previously [26,28]. In the case of silver layers, there are additional bands in the permittivity spectrum, observable for both Se and Te interlayers, similar to the segregation-induced bands reported for Ge interlayer [12,25–28]. For Te, the band is centered at 510 nm, while for Se it is at 470 nm. Both bands are much smaller than the Ge-induced band, although the time period in which the semiconductor atoms segregated (from the deposition of the sample to the ellipsometric measurement) is very similar. There are several reasons for this. The crystal size in metal layers deposited on Se and Te films is greater than for the Ge-wetted layers and so the probability of a grain being decorated by the semiconductor atoms to the same extent is lower [28]. Secondly, poor adhesion of the plasmonic metals to both Se and Te probably slows the segregation process down. In the case of the silver sample deposited with the Se interlayer, the segregation-induced band is barely noticeable. That is most likely due to the fact that a thin Se film has a glass- or even air-like permittivity and this is in agreement with an effective medium approximation [36]. The peak at 326 nm in the electron energy loss function essentially detects the plasma frequency of the sample. As such, its relative value allows to compare the number of detected atoms which contribute to the plasma frequency [26]. If so, then the lower the value of the maximum of this peak, the fewer metal atoms and more semiconductor atoms are detected. Because each atom gives a stronger optical response at the surface (due to high absorption in the metal layer), than by comparing the value of the maximum of this peak, the rate of segregation towards the surface can be compared. The lower the value of this peak, the faster the segregation progresses. Thus both Te and Se have a similar segregation rate, and both have segregated less during the period of two weeks than Ge during the period of 10 days. This does not mean, however, that the segregation will not progress any further, particularly in the case of Te.

**Figure 4:**
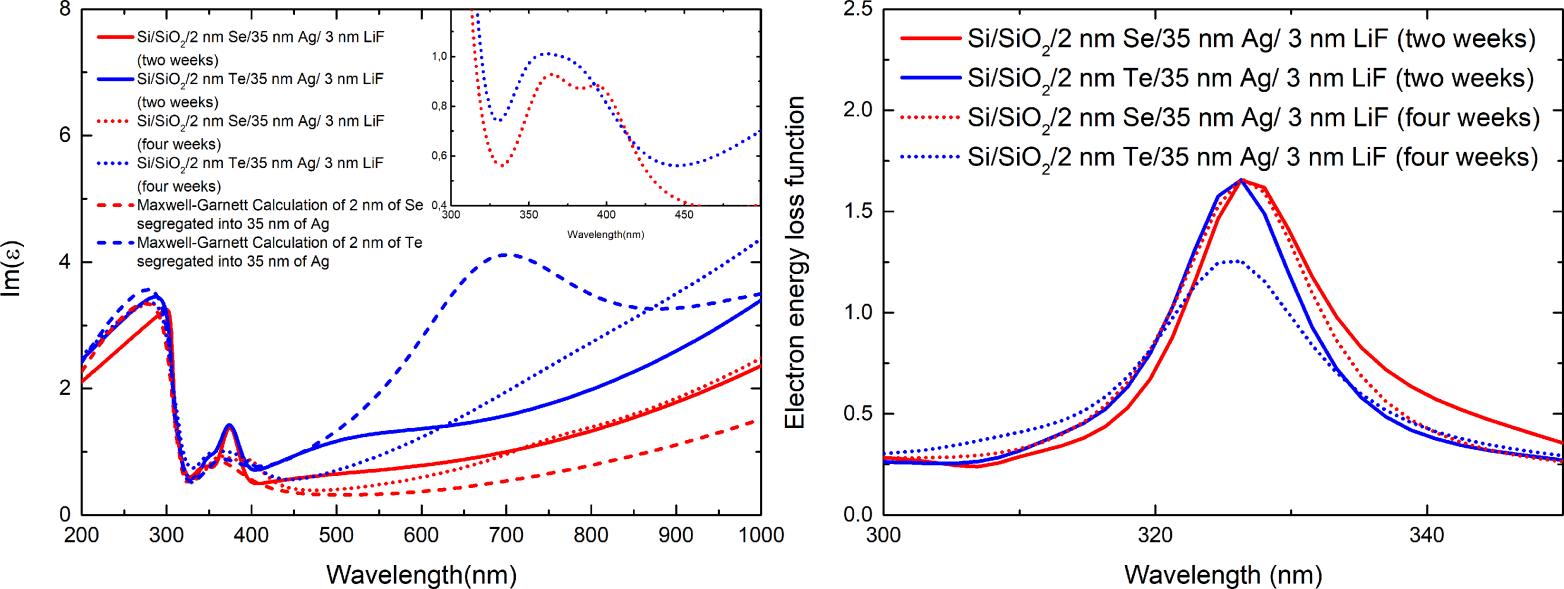
Left – Comparison of imaginary parts of the permittivity for 35 nm-thick Ag layers deposited on top of 2 nm-thick Se and Te films, measured either two or four weeks after deposition. Right – Electron energy loss function values for the same samples.

Figure 4 presents the permittivity curves and electron energy loss function for similar Ag layers deposited on top of 2 nm-thick Se and Te interlayers, but measured two weeks later (for a total of four weeks after deposition). Except for the band at 325 to 400 nm which will be discussed later, the permittivity of the silver layer deposited on top of selenium did not change much. Although the segregation-induced band did redshift from 470 to around 760 nm, it is still very weak and so the difference between the Im(ε) between measurements performed two and four weeks after sample fabrication is only 0.2 at 500 nm and gets smaller with wavelength. The loss function value at the maximum also stays the same, so we conclude that Se atoms do not migrate further into the Ag layer. The case of Te is however different. The most important difference in the permittivity spectra for samples measured two and four weeks after deposition is that for the latter, the contribution from the Drude term increases significantly, which results in higher values of Im(ε) in the long wavelength range. Since the Drude term is strictly connected to the sample resistivity, it suggests that after four weeks, much more Te atoms have migrated into the silver layer, forming a more uniform mixture. The segregation-induced band, although weakly noticeable to the eye due to high values of the Im(ε) originating from the Drude term, is still present. It is red-shifted to around 760 nm – this is not surprising since a similar effect is observed for Ge-wetted Ag films [26]. The new position of this peak is also in better agreement with the effective medium calculation, where this band is centered at 700 nm, although its intensity is much lower than the calculated one, which suggests that Te atoms no longer reside only in the Ag grain boundaries in the form of solute solution, but possibly also in the Ag grains, forming a nano-alloy. Moreover, after four weeks, the value of the loss function at the maximum is much lower. This implies that during the additional two weeks more Te atoms have segregated to the surface.

The poor wetting (or even dewetting) of silver by Se and Te has one more attribute. The intensity of the modified Lorentz band [37] centered at 275–300 nm decreased while the intensity of the bands from 325 to 400 nm increased. The latter are connected to the roughness of the layer (so the intensity would naturally increase with increased roughness) [26], but also to the interband transitions at the L-point in the Brillouin zone, while the former is connected to the interband transitions at the X-point in the Brillouin zone [38–39]. As the carriers at the X-point have a negative effective mass along one direction (X–Γ) and the carriers at the L-point have a negative effective mass along two opposite directions (L–X and L–K), the microstrain on metal grains would directly translate into changes in the intensity of the interband transitions and possible shifts in their energy, which is what we observe. It is also worth noting that the band at 325–400 nm clearly consists of two components, which confirms that this band has two origins: interband transitions at the L-point and surface plasmon excitations [26]. For the measurements performed four weeks after deposition, the intensity of this band is slightly lower, possibly due to reduced microstrain [21]. However, it still consists of two distinct components (see the inset of Figure 4 left), which confirms the double origins of this band.

Poor wetting of Ag layers by Se and Te certainly contributes to the high intensity of the band connected to the roughness and interband transitions at the L-point in the Brillouin zone. However, the fact that our samples have not been deposited on polished SiO_2_ substrates with low roughness as we have done previously, but rather on SiO_2_ evaporated on Si, might also influence the intensity of these bands.

The case of gold layers is quite similar to the silver ones – the interband transitions at 402 nm (X-point) and 322 nm (L-point) change intensity and slightly shift in energy when Au layers are deposited on Se or Te films. This indicates a microstrain on metal grains, which is confirmed by changes in the lattice constant of the metals films (see Table 1). We do not observe segregation-induced bands in the permittivity of the investigated gold layers, since Te does not segregate and the optical constant values of Se are very low [36]. The plasma frequency peak in the electron energy loss spectrum confirms that there is no Te segregation (the maximum value of the peak is very close to value for the pure gold layer) and indicates a slow Se migration to the surface (smaller value of the maximum than for the pure film), much slower than segregation of Ge [26,28].

It is worth noting, however, that although the microstrain on metal grains is the most obvious explanation of changes in the intensity of the interband transition peaks in the permittivity spectra of the investigated layers (since it is confirmed by the XRD and XRR results), we cannot fully exclude other mechanisms, such as nano-alloy formation, for example.

## Conclusion

We have fabricated ≈35 nm-thick silver and gold layers deposited on glass substrates with ultrathin Se and Te interlayers. XRR and XRD results showed that plasmonic layers exhibit poor adhesion to both semiconductor films, which results in high microstrain on metal grains and high surface roughness. This is confirmed by the abnormal ratio of the interband transition peaks in the permittivity spectra of the investigated layers. Despite that, thanks to the XPS measurements, high concentrations of Te and Se on the surface of Ag layers as well as Se on the surface of Au layer were detected. This indicates the occurrence of grain boundary segregation or diffusion of these semiconductors in the plasmonic thin films. The curve shape of the Te concentration in the Ag layer suggests the dominant role of segregation. The study of the electron energy loss spectrum allowed us to determine that both Se and Te migrate into the metal structure much slower than Ge.

## Experimental

The experimental details are similar to our previous works [12,28,36]. For most measurements, 2 in Si (111) substrates with a native SiO_2_ film were covered with an additional 100 nm-thick SiO_2_ layer, to avoid any influence from the pure silicon, which has been also reported to segregate in silver [29]. Then, ≈2 nm-thick Se and Te films were deposited on the SiO_2_ layer and were followed by 35 nm-thick layers of Ag or Au. For the XPS measurements, 20 nm-thick Ag or Au layers were deposited directly on SiO_2_ substrates, covered by 2 nm-thick films of Te or Se and then followed by another 20 nm-thick layer of Ag or Au. All of the samples were then capped with 3 nm-thick LiF films to avoid corrosion.

Se and Te films were deposited using a II–VI semiconductor growth chamber of a dual chamber molecular beam epitaxy (MBE) system delivered by SVT Associates. The substrates were kept at room temperature. The background pressure was below 5 × 10^−10^ Torr. The purity of sublimated ingots for both Te and Se was 7N. To avoid cross-contamination during the deposition of Te or Se only one Knudsen cell was kept at working temperature [39]. SiO_2_, Ag and Au, LiF layers were deposited from fabmate or tungsten crucibles using a PVD75 Lesker e-beam evaporator. The purity of the evaporation materials was 4N for both silver and gold, 5N for SiO_2_ and TIO_2_, 3N for LiF. SiO_2_ was evaporated at an average deposition rate of 5 Å/s, while silver and gold films were evaporated at an average deposition rate of 2 Å/s. LiF was evaporated at an average deposition rate of 1 Å/s. The deposition rate and total film thickness were monitored by two quartz weights inside the deposition chamber. Then, the film thicknesses were verified by a Dektak 6M stylus profiler. The pressure in the vacuum chamber was kept below 5 × 10^−5^ Torr during the whole deposition process. The crucible–substrate distance was 40 cm.

The X-ray reflectometry measurements were performed 3 days after the deposition of the samples using a Bruker Discover D8 X-ray diffractometer working with a Cu Kα line source of wavelength 0.154 nm; the diffraction signal was recorded with a point scintillation detector. The monochromatic parallel beam was formed by crossed parabolic Goebel mirrors. The data analysis was based on finding the proper electron density profile for which with XRR generated data matched the experimental one. To compare our data with data on silver and gold layers wetted with germanium films, in the XRR model, we have split the metal layer into 10 sublayers and set the exponential change in the density of each sublayer [12,28]. Data fitting was performed using Leptos 4.02 software package provided by Bruker. The electron density was simulated by a box type function. The thicknesses of the Se and Te wetting films were fitting parameters (the density of this film was fixed) and for all samples, the fit equated 2 ± 0.5 nm. The optical thickness of the Ag and Au layers were fitted for the samples without the wetting films fabricated in the same processes, and then fixed for all other samples, while the density was left as a fitting parameter for all samples. The thickness and density of the LiF protective films were fitted for the samples without wetting films, and then fixed for all other samples. The wetting films/metal and metal/LiF interface roughness were left as fitting parameters. More information about XRR modeling can be found in [40] and references therein.

The wide-angle X-ray diffraction (XRD) measurements were performed in transmission mode using a Bruker Discover D8 GADDS system. The system works with Cu Kα X-ray source. The X-ray patterns are recorded with a 2D Vantec 2000 detector. For precise diffraction angle measurements, a Bruker Discover D8 system was also used, but the measurements were performed in reflection geometry in θ–2θ scans. The X-ray signals were recorded with a 1D Vantec-1 detector. The width and position of the signals were analyzed with TOPAS software. Since all other diffraction peaks in the XRD spectrum were extremely weak for both silver and gold layers, the average size of the gold grains was then calculated by fitting the Gaussian profile to the dominant diffraction peak at 38.2°, which corresponds to 111 orientation of grains with respect to the c-axis. Then the full width at half maximum (FWHM) parameter of the fitted Gaussian profile was used in the Debye–Scherrer formula:

[1] $$d=\frac{0.89\lambda }{\text{FWHM}\cdot \cos\theta }$$

where *d* is the average grain size, λ is the incident wavelength (in this case 0.154 nm) and θ is the Bragg diffraction angle. The lattice constant was then derived from the position of the fitted Gaussian profile.

Ellipsometric azimuths of the fabricated samples were measured 13 days after the deposition of the samples in the UV–vis–mid-IR spectral range (0.06–6.5 eV) for three angles of incidence (65°, 70° and 75°) using two instruments: V-VASE (J.A.Woollam Co., Inc.) in the UV–vis–NIR and Sendira (Sentech GmbH) in the mid-IR. The complex dielectric function of effective Ag layers with segregated Te or Se atoms was extracted using a layered model of the samples. The permittivity was then interpreted in terms of the Lorentz, Drude–Lorentz and modified Lorentz [37] oscillator models. The electron energy loss function (LF) was calculated from the permittivity values using the following formula: LF = −Im(ε^−1^), where ε is the complex permittivity of the layer.

The XPS measurements were performed 20 days after the deposition at base pressure ≤2 × 10^−10^ mbar. Monochromatic radiation from Al Kα source (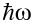 = 1486.6 eV) was used to excite photoelectrons, and the incidence angle was 55°. Photoemission spectra were recorded using a VG Scienta R3000 hemispherical analyzer oriented perpendicular to the sample surface. The XPS data were recorded for all of the elements found at the surface with the energy resolution set to 100 meV (with the exception of lithium). The XPS data recordings were interlaced by Ar-ion etching in order to study the chemical composition of subsequent sublayers. The energy of Ar ions was 4 keV and the incidence angle was 69°. An ion beam scanned an area of 4 × 4 mm in order to etch the analyzed surface homogeneously. The concentration of elements was estimated by fitting the most intense peaks to Gauss–Lorentz shapes by using Casa XPS software.

The effective medium calculations were performed using the Maxwell–Garnett formula [41–42]:

[2] $$\varepsilon_{\text{eff}}=\frac{\varepsilon_{\text{m}}\left(2f\frac{\varepsilon_{\text{i}}-\varepsilon_{\text{m}}}{\varepsilon_{\text{i}}+\varepsilon_{\text{m}}}+1\right)}{1-f\frac{\varepsilon_{\text{i}}-\varepsilon_{\text{m}}}{\varepsilon_{\text{i}}+2\varepsilon_{\text{m}}}}$$

where ε_eff_ is the effective permittivity of the system, ε_m_ the permittivity of the medium (Ag layer), ε_i_ the permittivity of the inclusion (Te or Se atoms), and *f* is the inclusion fill factor.
